# High-resolution early warning system for human Puumala hantavirus infection risk in Germany

**DOI:** 10.1038/s41598-024-60144-0

**Published:** 2024-04-26

**Authors:** Orestis Kazasidis, Anke Geduhn, Jens Jacob

**Affiliations:** 1 Institute for Epidemiology and Pathogen Diagnostics, Rodent Research, Julius Kühn Institute (JKI) – Federal Research Centre for Cultivated Plants, Toppheideweg 88, 48161 Münster, Germany; 2 Laboratory for Health Pests and Their Control, German Environment Agency, Corrensplatz 1, 14195 Berlin, Germany

**Keywords:** Ecological epidemiology, Ecological modelling

## Abstract

The fluctuation of human infections by the Puumala orthohantavirus (PUUV) in Germany has been linked to weather and phenology parameters that drive the population growth of its host species. We quantified the annual PUUV-outbreaks at the district level by binarizing the reported infections in the period 2006–2021. With these labels we trained a model based on a support vector machine classifier for predicting local outbreaks and incidence well in advance. The feature selection for the optimal model was performed by a heuristic method and identified five monthly weather variables from the previous two years plus the beech flowering intensity of the previous year. The predictive power of the optimal model was assessed by a leave-one-out cross-validation in 16 years that led to an 82.8% accuracy for the outbreak and a 0.457 coefficient of determination for the incidence. Prediction risk maps for the entire endemic area in Germany will be annually available on a freely-accessible permanent online platform of the German Environment Agency. The model correctly identified 2022 as a year with low outbreak risk, whereas its prediction for large-scale high outbreak risk in 2023 was not confirmed.

## Introduction

Puumala orthohantavirus (PUUV) occurs in large areas in Europe^1^ and can cause severe disease (*nephropathia epidemica*) with kidney failure in humans. About 150,000 to 200,000 people are hospitalized every year because of severe symptoms of PUUV infection^2^. Symptoms develop after an incubation period of ca. two weeks and include headache, abdominal pain, back pain, fever, and renal failure that can require dialysis^3^ but case fatality is low (0.1%)^4^.

PUUV is transmitted to humans by the PUUV host species bank vole (*Myodes glareolus*)^1^. Bank voles inhabit forests, hedges and peri-urban habitats from Spain to central Russia and from northern Finland to southern Italy^5^. In Central Europe, they reach population densities of > 100 individuals/ha^6^. PUUV-infected bank voles shed PUUV in excreta to the environment where it can persist for several weeks^7^. PUUV transmission to humans is mainly via the environment. The virus remains infectious in the environment for several weeks and can be transmitted via inhalation of aerosols contaminated with virus excreted by bank voles^8^.

The risk of human PUUV-infection and PUUV-incidence depends on the population density of infected bank voles^9–11^. Especially multi-annual outbreaks of bank vole populations provide the conditions for frequent human infections. In Central Europe, the occurrence of population outbreaks of bank voles is regulated bottom-up by the availability of food^12^. This is similar to the regulation of other rodent outbreaks via high quality food, including house mice (*Mus dometicus*) in New Zealand and wood mice (*Apodemus sylvaticus*) in Norway that depend on seed mast^13,14^. Such high quality food for bank voles are the seeds of common beech trees (*Fagus sylvatica*). Beechnuts are a primary food source for bank voles and their supply has been linked to the growth of rodent populations in Central Europe^10,15,16^. When intensive beech seed production is synchronized over large areas^17^, leading to so-called mast years, bank vole survival and (winter) reproduction is increased^18^, boosting population growth in the year after the beech mast. Beech mast depends on weather conditions^19,20^ (resource matching hypothesis) and highly correlates with temperature two years before the beech mast (negatively) and in the year prior to beech mast (positively)^21^. The correlation of weather, beech mast and population dynamics of bank voles has be recognized previously^4^ identifying time lags in weather effects^22,23^ on human PUUV-infection risk and speculation about the impact of precipitation^4^ in several European countries.

Weather conditions alone fail to explain all beech mast variability^24^ and other selection hypotheses have been suggested, such as wind-pollination efficiency and predator satiation^25^. Regardless, masting is expected to matter not only for bank vole population dynamics but also for the epidemiology of PUUV. Weather effects can act directly on the virus (e.g. stability in the environment)^8^ or indirectly through the influence of vegetation (other than beech, e.g. hornbeam and oak) and thus the food availability of the bank vole^26^.

Since these parameters occur long before the population increase of bank vole population density and human PUUV-infections, they can be used to predict the risk of human PUUV-infections. Previous attempts to predict human PUUV-infection risk include approaches using bank vole population/infection data^9,27,28^, but approaches focusing on extrinsic parameters are rare^23^. In Germany, PUUV is mainly endemic in the west and southwest. Two models for PUUV-prediction in Germany have been developed previously^29,30^, but both are restricted to particular regions.

Currently, there is no vaccination against PUUV or any other relevant orthohantavirus available and there is no PUUV-specific treatment. Predictions based on extrinsic parameters such as weather and seed mast might be coarser than fine-tuned data from monitoring the rodent host^9,28^. However, they take advantage of the time lag present between changes in extrinsic parameters and effects on bank voles, PUUV-prevalence and human incidence. This results in an increased lead-up time to inform risk groups, the general population and public health authorities about the risk of a PUUV-outbreak at an early stage, so that preventive measures to avoid infection can be taken in time^31^.

The aim of this study was to develop a prediction model that is suitable for the entire endemic area of PUUV in Germany with sufficient predictive power. Risk maps resulting from the prediction are made available on a permanent online platform^32^ which will be updated annually in the fall of each year for the following year. In contrast to previous, more academic exercises to link intrinsic and extrinsic parameters to human PUUV-infection risk, this approach is hoped to result in translation of forecast results in disease prevention.

## Results

### Selection of predictors

**Figure 1:**
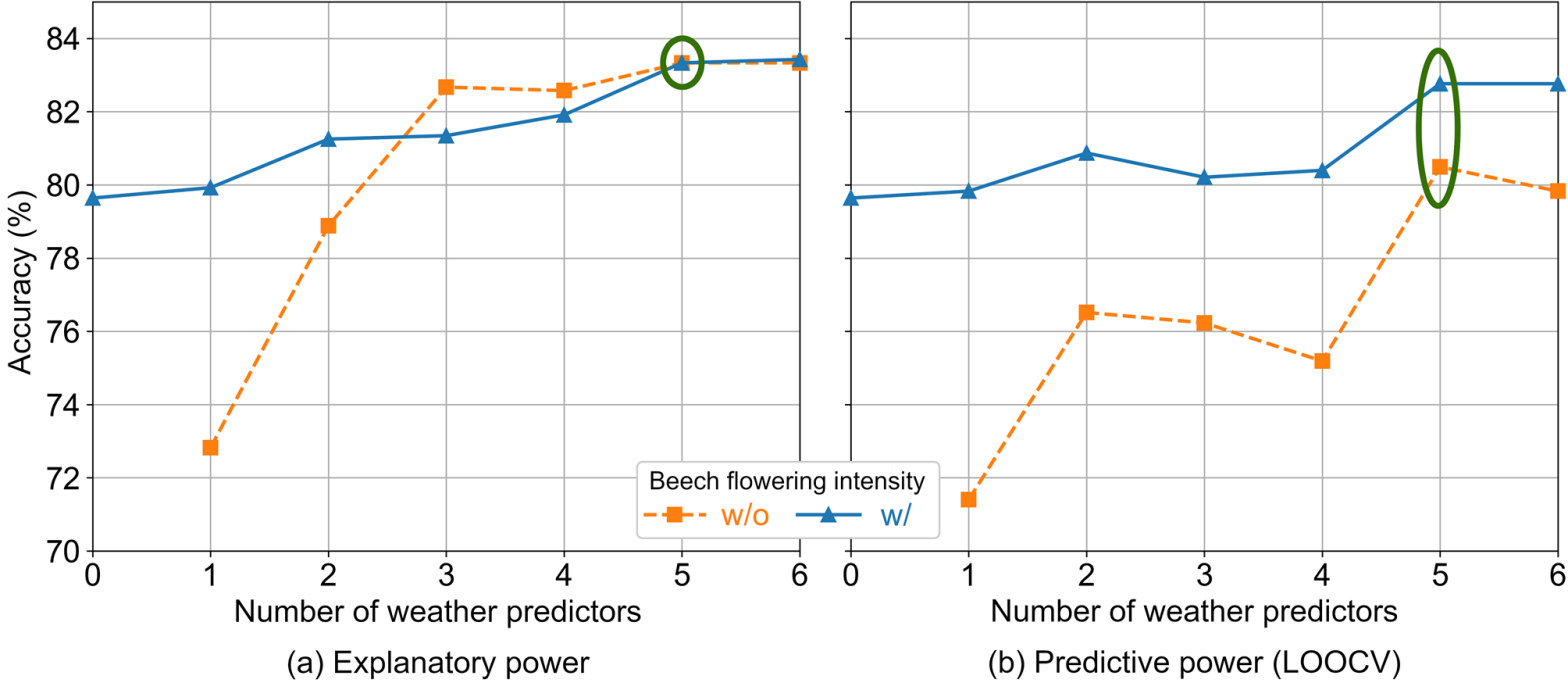
(**a**) The mean accuracy over all districts of the best model on the training dataset for different numbers of predictors. This is a metric for the explanatory power of each model. (**b**) The mean accuracy over all districts for each individual year after a leave-one-year-out cross-validation (LOOCV) for different numbers of predictors. This is a metric for the predictive power of the method. The orange dashed lines with the rectangular markers show the models without the flowering intensity from the previous year (w/o). The blue solid lines with diamond markers show the models with the flowering intensity from the previous year (w/). The green ellipses denote the optimal models either without or with the flowering intensity from the previous year, which are the final models.

Our model is based on a support vector machine that classifies the annual outbreak risk at the district level. We selected the combination of predictors that achieved the optimal performance from a total of 149 available variables: the beech flowering intensity of the two previous years, and 7 monthly weather variables from two years before and from the previous year until September. From all 2-variable combinations, the best performance was achieved with combinations of weather conditions in April and September from two years before, and in September of the previous year. Figure 1 shows the models with the best performance for up to 6 weather predictors. The model performance on the training dataset 2006–2021 was used to assess the explanatory power (Fig. 1a). The predictive power of the method was assessed using the result of a leave-one-out cross-validation (LOOCV) (Fig. 1b). The explanatory power increased with the inclusion of further predictors. The increase is expected to slow until the accuracy reaches its maximum value and fluctuates around it. On the other hand, the predictive power is not an increasing function of the number of predictors and is expected to have several local maxima. This means that the inclusion of additional predictors does not always lead to increased accuracy.

We selected the models with five (5) predictors with or without the flowering intensity from the previous year, shown with green ellipses in Fig. 1 and discussed in the following section. For the subsets without the flowering intensity from the previous year, the selection was unambiguous because the predictive power even slightly decreased at a subset size of 6. The predictive power may increase again for a larger number of predictors, but we limited the search at up to 6 weather predictors due to limited computing power. For the subsets with the flowering intensity from the previous year, the predictive power remained constant for subset sizes of 5 and 6. Therefore, we chose the subset with the smallest size. Moreover, the additional (sixth) weather predictor was the soil moisture from the previous June, which correlated strongly with the soil moisture from the previous May that was already included in the model (0.65 Pearson correlation coefficient, p ≪ 0.001) and will continue to correlate in the future. A low model complexity, especially without strongly correlated predictors, is important to avoid overfitting and to escape the “curse of dimensionality”.

By observing the explanatory power alone (Fig. 1a), we would conclude that the flowering intensity of the previous year is not required, because the accuracy is similar for subset sizes ≥ 3. However, the difference became apparent in the predictive power (Fig. 1b), because the mean annual accuracy of the models with the flowering intensity of the previous year was always higher. The difference between the averages of the two sets was statistically significant (one-way ANOVA with p < 0.05). Including the flowering intensity of the previous year is expected to increase the predictive power of any subset of predictors. The model with only the flowering intensity of the previous year already had an accuracy of 79.6% in the LOOCV. A model with only weather variables had to include at least five predictors to achieve this level of accuracy. This demonstrates the predictive power of the flowering intensity of the previous year. Although the weather variables correlate with the flowering intensity of the following year, including them results in a more robust model.

### Optimal models

The optimal model included five weather predictors and the flowering intensity of the previous year. The predictors from two years before were the maximum air temperature in April (Tmax_4), the precipitation in September (Pr_9), and the mean air temperature in November (Tmean_11). The predictors from the previous year were the soil moisture in May (SM_5), the soil temperature in September (ST_9), and the flowering intensity. Table 1 lists the coefficients of the model, which represent the coordinates of a vector orthogonal to the hyperplane separating the low and high outbreak risks. Therefore, a positive coefficient means that, if the values of all other predictors remain constant, an increased value of the specific predictor would move the data point closer to the hyperplane, or even to its opposite side (if the point indicated a low risk of outbreak), or further from the hyperplane (if the point already indicated a high risk of outbreak). The positive coefficients are those of the precipitation in September from two years before, and of the flowering intensity and of the soil temperature in September from the previous year.

**Table 1:** The coefficients of the support vector machine classifier for the optimal model including the beech flowering intensity.

Constant	Tmax_4 year-2	Pr_9 year-2	Tmean_11 year-2	SM_5 year-1	ST_9 year-1	Flowering intensity year-1
− 3.5083	− 0.7036	0.0102	− 0.1515	− 0.0889	1	1.4449

The coefficients were scaled to the value of ST_9 from the previous year and rounded to four decimal digits.

**Figure 2:**
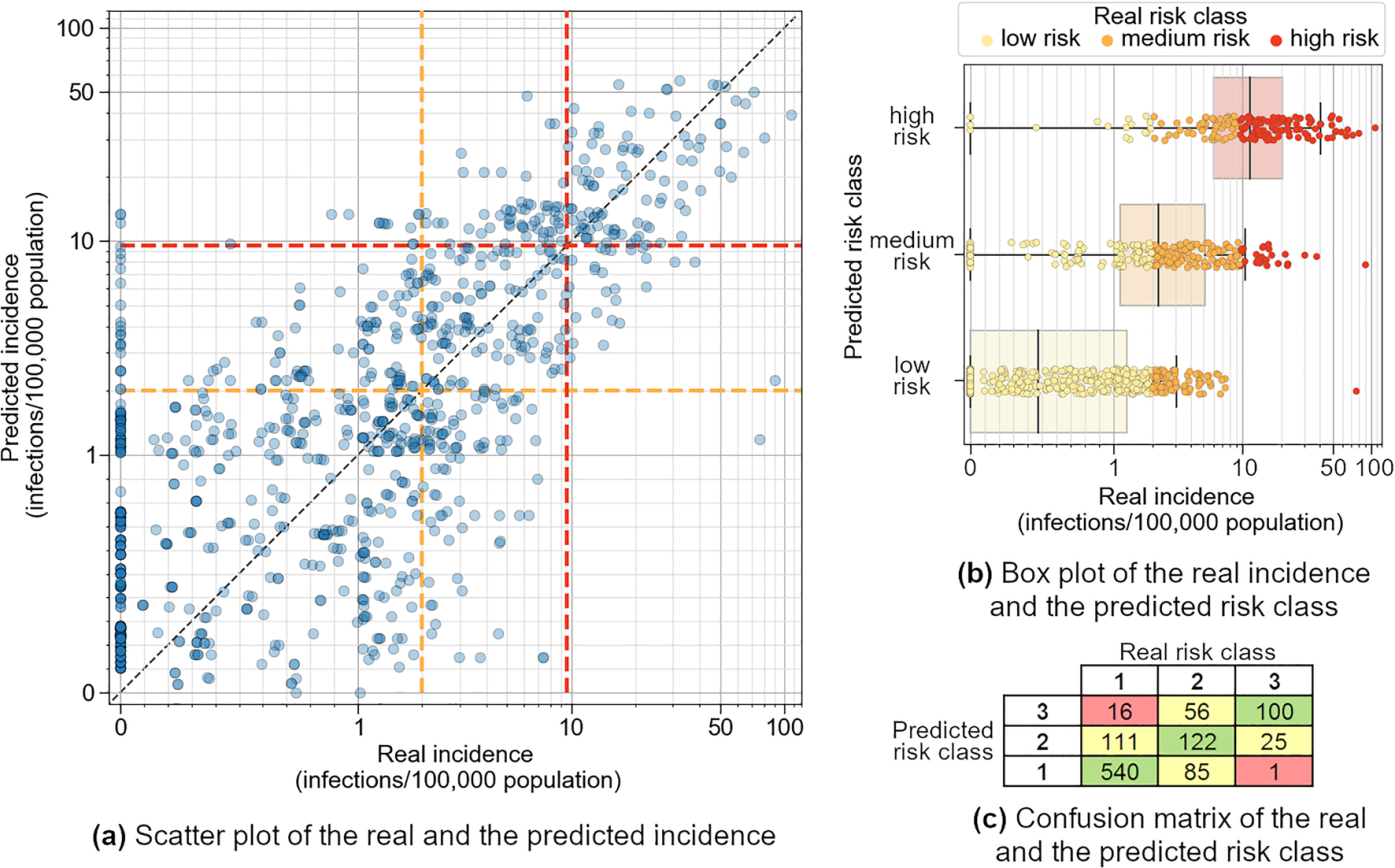
The results of the leave-one-year-out cross-validation in 2006–2021 for the optimal model with five weather predictors and the flowering intensity of the previous year. (**a**) The real incidence plotted over the predicted incidence. For a clear representation, both axes are linear in the range [0, 1] and logarithmic in the range [1, 120]. The colored dashed lines denote the incidence thresholds for defining the risk classes: 2 and 9.5. The horizontal lines define the predicted risk classes and the vertical lines define the real risk classes. (**b**) Boxplots for the predicted risk classes and the real incidence. The box for each predicted risk class contains the middle 50% of the incidence values. The thick line inside the box represents the median of the data points. The boundaries of the whiskers were set to the 1.5 of the interquartile range (IQR). The color of each box corresponds to the predicted risk class. The color of each data point corresponds to the real risk class. (**c**) The confusion matrix for the risk classes. The cell coloring supports the clarity. The green cells would ideally contain all values and correspond to the line y = x of the plot in (**a**). The yellow cells indicate one-class deviations, whereas the red cells indicate two-class deviations. The cells above the green diagonal are overestimations of the risk class, and the cells below it are underestimations of the risk class. In all plots, the vertical axis represents the prediction and the horizontal axis represents the real outcomes. Therefore, the plots in (**b**) and (**c**) appear rotated in comparison to their most common orientation.

**Figure 3:**
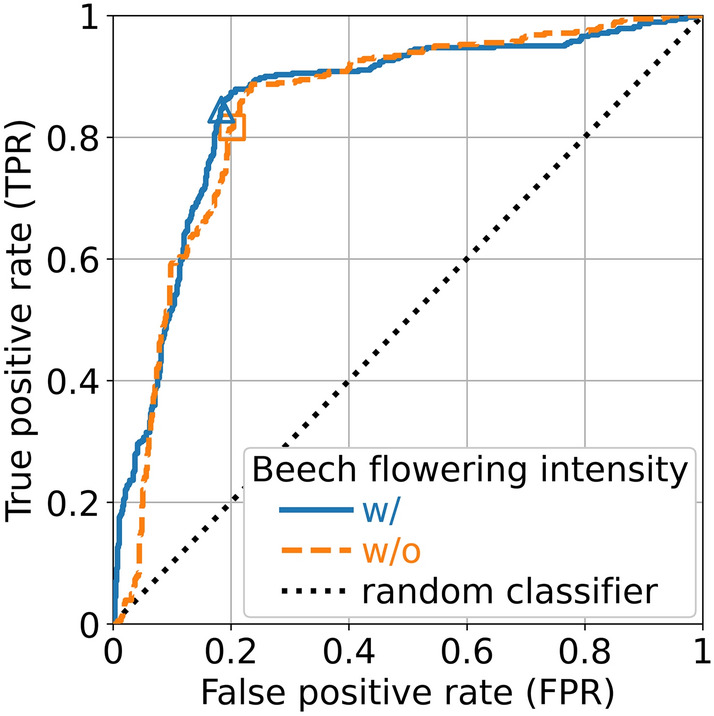
The ROC curves for the two optimal models, as calculated from the real outbreak risk and the distance of the data points from the hyperplane of the support vector machine classifier, using the result of the LOOCV. The blue solid line shows the ROC curve for the model with (w/) the flowering intensity from the previous year (AUC = 0.86), whose operating point for zero threshold was at FPR = 0.184 and TPR = 0.845 (diamond marker). The orange dashed line shows the ROC curve for the model without (w/o) the flowering intensity from the previous year (AUC = 0.85), whose operating point for zero threshold was at FPR = 0.201 and TPR = 0.816 (rectangular marker). For reference, the black dotted line shows the ROC curve for a random classifier (AUC = 0.50).

The model had a mean annual accuracy of 82.8% for the outbreak risk in 16 years (LOOCV) across all 66 selected districts, i.e., a total of 1056 data points (Fig. 2a). The annual accuracy for the outbreak risk had a maximum of 100%, a minimum of 42.4%, and a standard deviation of 15.4%. There were 246 observations with zero real incidence but nonzero predicted incidence up to 15 infections per 100,000 population, 24 of which resulted from an overestimation of the outbreak risk. The most extreme incidence deviations were in 2007 for LK Heidenheim and LK Zollernalbkreis, with a real incidence of 90.2 and 76.5 respectively (Fig. 2a), both resulting from an underestimation of the outbreak risk. The median values of the real incidences for each predicted risk class were well within the ranges defined by the incidence thresholds of 2 and 9.5: 0.5 for the low-risk class, 2.2 for the medium-risk class, and 11.5 for the high-risk class (Fig. 2b). The coefficient of determination for the incidence was 0.457 (Fig. 2a), and the mean annual accuracy for the risk classes was 72.2% (Fig. 2c). The area under the curve (AUC) calculated from the receiver operating characteristic (ROC) curve (Fig. 3) was 0.86.

The collection of flowering intensity data is voluntary and may not be assured in the long term. Therefore, we also selected the optimal model without the flowering intensity to ensure that the prediction model will remain functional even without the flowering intensity. The optimal model without the flowering intensity included five weather predictors. The predictors from two years before were the maximum air temperature in April (Tmax_4) and the sunshine duration in September (SD_9). The predictors from the previous year were the soil moisture in April (SM_4), and the soil temperatures in June (ST_6) and in September (ST_9). Table 2 lists the coefficients of the model. Comparing Tables 1 and 2, the common predictors of the two models are the maximum air temperature in April (Tmax_4) from the previous year and the soil temperature in September (ST_9) from the previous year. The coefficients of both predictors have the same signs for both models, negative and positive, respectively. The predictors from the additional common year-month combination are Pr_9 and SD_9 from two years before, with positive and negative coefficients, respectively. Their reverse relationship with outbreak risk was validated from the negative correlation between the two predictors (− 0.41 Pearson correlation coefficient, p ≪ 0.001).

**Table 2:** The coefficients of the support vector machine classifier for the optimal model with only weather variables.

Constant	Tmax_4 year-2	SD_9 year-2	SM_4 year-1	ST_6 year-1	ST_9 year-1
4.7835	− 0.3727	− 0.0164	− 0.0590	− 0.4313	1

The coefficients were scaled to the value of ST_9 from the previous year and rounded to four decimal digits.

The model had a mean annual accuracy of 80.5% for the outbreak risk in 16 years (LOOCV) across all 66 selected districts. The annual accuracy for the outbreak risk had a maximum of 97.0%, a minimum of 39.4%, and a standard deviation of 16.6%. The coefficient of determination for the incidence was 0.418 and the mean annual accuracy for the risk classes was 71.1%. The area under the curve (AUC) calculated from the ROC curve (Fig. 3) was 0.85.

For the final models, the validation and test datasets were omitted, and all observations were included in the training dataset, because all past years are equally important. The two optimal models had comparable risk class accuracy: 83.2% for the model with the flowering intensity of the previous year and 83.3% for the model with only weather variables. The confusion matrices of both models were diagonally dominant, with 178 overestimations and 84 underestimations out of 1,056 data points for the model with the flowering intensity; and 183 overestimations and 78 underestimations for the model with only weather variables. Both models assigned a high outbreak risk to all districts in 2010, 2012, 2017, and 2019; and a low outbreak risk in 2008, 2009, 2011, 2013, 2016, 2018, and 2020. In addition, the optimal model with the flowering intensity assigned a low outbreak risk to all districts in 2006 and 2014; whereas the model with only weather variables additionally assigned a high outbreak risk to all districts in 2015 and 2021. The predictions for the remaining years consisted of a mixture of low and high outbreak risk in each model.

### Prediction and validation for 2022

Using weather variables from 2020 and 2021, and flowering intensity values from 2021, we generated the prediction for 2022. The two selected models assigned a low predicted outbreak risk to all districts, resulting in the same predicted incidence values and predicted risk classes. Both models predicted an incidence > 2 infections per 100,000 population for only 5 districts: LK Göppingen, LK Heidenheim, and LK Sigmaringen from BW, and LK Freyung-Grafenau and LK Main-Spessart from BY. The remaining districts had a predicted incidence < 2.

In 2022, a total of 49 PUUV-infections were reported. The highest reported incidence was 2.55 in LK Freyung-Grafenau (BY) with 2 infections. The district of Böblingen (BW) reported 4 infections and the districts of Karlsruhe (BW) and Wesel (NW) each reported 3 infections. A total of 8 PUUV-infections (16% of the total annual cases) were reported in districts not included in the model: 2 cases in SK Berlin that are probably connected to an infection outside the district of residence, and another 6 districts with a single reported case each. The prediction accuracy for both models was 100% for the outbreak risk and 93.9% for the risk classes, with 62 correct predictions and 4 overestimations for districts in BW (LK Göppingen, LK Heidenheim and LK Sigmaringen) and BY (LK Main-Spessart).

### Prediction and validation for 2023

Using weather variables from 2021 and 2022, and flowering intensity values from 2022, we generated the prediction for 2023. The two selected models assigned the same prediction to 14 districts in BW and BY with low outbreak risk, and to 11 districts in NI and NW with high outbreak risk. A relatively high number of human PUUV-infections was predicted by both models for the following districts in 2023: LK Borken, LK Coesfeld, LK Emsland, LK Grafschaft Bentheim, LK Osnabrück (combined with SK Osnabrück), LK Recklinghausen, LK Steinfurt, LK Warendorf, LK Wesel, SK Köln (Cologne) and SK Münster.

The model with the flowering intensity predicted that 41 additional districts in Central and Southern Germany will have a high outbreak risk, whereas the model with only weather variables predicted no outbreaks there. This difference propagated to the risk classes. The difference between the two models was caused by a sign change in the distances of those 41 observations from the hyperplane defined by the support vector machine classifier. These sign changes can be regarded as an offset of the data cluster that approaches or even exceeds the hyperplane, demonstrating the importance and dominance of the flowering intensity for the model.

In 2023, a total of 139 PUUV-infections were reported (date of query: 2024-02-09)^33^. The highest reported incidence was 12.76 in LK Freyung-Grafenau (BY) with 10 infections. The district of Osnabrück (combined LK and SK Osnabrück, in NI) reported 14 infections (an incidence of 2.67) and the district of Steinfurt (NW) reported 11 infections (an incidence of 2.45). A total of 28 PUUV-infections (20% of the total annual cases) were reported in districts not included in the model: 4 cases in LK Minden-Lübbecke (NW); 2 cases in each of LK Cloppenburg (NI), LK Rhein-Erft-Kreis (NW) and the district of Aachen (NW); and another 18 districts with a single reported case each.

The reported cases indicated high outbreak risk only in three districts: LK Freyung-Grafenau (BY), LK Steinfurt (NW), and LK Emsland (NI). The cases in all other districts indicated low outbreak risk. For example, the mean number of infections in Osnabrück in the 5 previous years with high outbreak risk was 62.6, with a minimum of 27 in 2017. This number is significantly larger than the 14 reported cases. The prediction was uncertain only for districts in Central and Southern Germany, due to the different result from the two selected models. Nevertheless, both models agreed that endemic areas in Northern Germany would have high outbreak risk. The prediction accuracy for the model with the flowering intensity was 22.7% for the outbreak risk (50 overestimations and 1 underestimation in 66 observations), and 27.3% for the risk classes. In comparison, the prediction accuracy for the model with only weather variables was 84.8% for the outbreak risk (9 overestimations and 1 underestimation in 66 observations), and 80.3% for the risk classes. Both models, but especially the model with the flowering intensity, overestimated the infection risk in 2023. Any modification of the reporting process of the PUUV-cases cannot sufficiently explain the discrepancy. In case the annual predictions of the model with the flowering intensity systematically underperform in the future, a plausibility check of the flowering intensity data is deemed necessary.

### Online platform

The final prediction model including the beech flowering intensity was used to generate a risk map for the outbreak risk for all German districts where PUUV is endemic. Furthermore, historic values of human PUUV-infections were used to predict incidence values in outbreak and non-outbreak scenarios and were displayed in a second risk map. In a third step, three risk classes (low, medium, high infection risk) were derived from the predicted incidence values and presented in a third risk map. Since May 2023, the three risk maps are available on the website of the German Environment Agency^32^. The risk prediction will be produced annually in fall using the developed model and published on the website. In addition to the PUUV-prediction, the interactive map also contains historical data on outbreak risk, risk class and PUUV-incidence. The risk map is freely accessible to both general public and health care authorities. The prediction for 2024 can be found online^32^.

## Discussion

This is the first prediction model for human PUUV-infection risk across the whole of Germany. It is used as an early warning tool^32^ as one of the elements in a decision-support system of public health authorities whether or not to issue warnings to general practitioners and risk groups. Furthermore, citizens or vacationers in PUUV-endemic districts can inform themselves about the risk of an outbreak and contact a doctor in case of symptoms after possible exposure.

The model combines low complexity with high predictive power. Its predictors can provide insights into the ecological, biological and social mechanisms that drive the human PUUV-infections, which in turn can be applied to refine the model in the future. The model can be extended to presently non-endemic areas of Germany where PUUV may become endemic in the future. Such a method based on weather and phenology parameters is also likely to be applicable to other regions in Central and Western Europe that have similar ecological parameters, such as the Netherlands, Belgium, Denmark, and France. Presumably, other parameters will be relevant for predicting PUUV-infections in different regions of the world, but the type of modelling could be used for other countries as well.

Our method is based on the outbreak risk, which we first introduced in a previous study^34^. Based on this quantity that precedes the prediction of incidence and risk class, each district will either have an outbreak or not in each year. In this study, we expanded the analysis in three steps: (1) the beech flowering intensity was added in our predictors’ pool, (2) the heuristic method for selecting the optimal predictors was extended to subset sizes up to six weather predictors plus the beech flowering intensity of the previous year, and (3) an incidence prediction was generated. In addition, the prediction model was validated via cross-validation for all 16 years in our original training dataset (2006–2021).

Although our method may not find the global maximum with respect to a given performance criterion, it avoids including highly correlated variables that could lead to overfitting, and it is bound to have high sensitivity and precision. Depending on the used performance criterion and the class weights, a model with a different subset of predictors may be chosen as optimal.

We chose a support vector machine (SVM) classifier with a linear kernel as the classification method, after observing the distribution of the data points with low and high outbreak risk in relation to two and three predictors^34^. However, it is not certain that the two classes are perfectly linearly separable. Our approach remains valid provided that the human PUUV-infections fluctuate between two extremes. A third intermediate class for outbreak risk would provide better accuracy, but its distinction was not possible with the currently available data. An adjustment of the regularization parameter C and the class weights of the SVM, and modelling with a radial basis function (RBF) kernel showed no significant improvement in the predictive power.

Our method does not consider any quantitative effects of the predictors on incidence or infections. Instead, the incidence is assumed to fluctuate between two extreme values. The uncertainty in the calculation of the incidence depends on the estimation of its expected value from the historical data. An adjusted calculation of the expected incidence value could be carried out in individual cases, taking into account the actual effects, changes in the reporting system or in the habitat of the rodents, and the relevance of the publication of these prediction results at the district level.

Part of the method inaccuracy is due to the unsupervised clustering analysis of the incidence, which was used to assign the labels. For example, a horizontal shift of the probability distribution for the class of low outbreak risk would lead to wrong labels. In this example, an increased incidence value, which may actually indicate a new level for non-outbreak years, could be attributed to the class of high outbreak risk. This can be caused by a significant change in the reporting system that would result in the number of reported infections being closer to the number of actual cases, i.e., a lower number of unreported cases. We suspect that many of the model’s underestimations in NI and NW over the past few years were not due to a local outbreak, but rather due to mislabeling^34^. Such inaccuracy can only be reduced by manual data cleaning for the false negative data points, i.e., by closely examining any incorrect model classifications and appropriately correcting their labels. However, this approach would be arbitrary.

The remaining method inaccuracy is due to phenomena not accounted for in the models, and to probabilistic effects that can lead to an outbreak even if the predictor values do not indicate it and vice versa. The weather variables from the prediction year cannot be used for a prediction model, but still influence bank vole populations^35,36^, human activities^37^ and their interaction.. The same applies to the weather variables after September of the previous year. In addition, it can be expected that all preventive measures will influence and specifically reduce the future risk of infection. These measures include the establishment of this systematic prediction model. This should be taken into account in any future validation or model update and it can be used to assess the effectiveness of preventative action.

Predictor pairs including weather variables after September of the previous year did not show superior performance. Therefore, we limited the predictors until September of the previous year in order to provide as much lead-time for the early warning system as possible. Indeed, the last predictor was from the previous September, which provides sufficient time to raise awareness in health authorities, risk groups and medical practitioners about human PUUV-infection risk.

The weather predictors from two years before were universal: the maximum and the mean air temperature, the precipitation, and the sunshine duration. They can account for weather effects on the beech seed production of the following year as well as for their direct influence on bank vole population growth. On the other hand, weather predictors from the previous year are soil-specific: the soil moisture and the soil temperature.

Our previous simplified model^34^ included only three weather predictors. The soil temperature of the previous September was also included in the optimal set of predictors of this study. However, the total sunshine duration in September of two years before was replaced by the precipitation, and the soil temperature in April of two years before was replaced by the maximum air temperature. The substitutions are explained by the better synergy of the updated variables from the same months, when increasing the number of predictors.

The optimal set of predictors included the beech flowering intensity of the previous year, whose spatial distribution reflects the mast variability in each year. Its inclusion accounts for spatially varying effects that cannot be explained by weather conditions alone. The fact that the models including the beech flowering intensity have superior predictive power in comparison to models with only weather variables is a testament that the flowering intensity is not solely governed by weather conditions, as the resource matching hypothesis indicates.

Although the flowering intensity in late spring and early summer is a good proxy for masting, increased flowering does not always translate to increased seed production^38^. Data for the beech seed production published by the Federal Office for Agriculture and Food^39^ are neither uniform nor harmonized among either the regions of provenance or the federal states. On the other hand, beech fructification data published by the federal forest departments or the corresponding ministries for agriculture are only available at the level of the federal states (13 area-states and 3 city-states), which is a lower spatial resolution than the level of the regions of provenance, which is the resolution of the beech flowering intensity data. Both the seed production data and the fructification data become available either late in the year or around the spring of the following year and therefore, they cannot serve as predictors for timely forecast. However, they could be used for validation purposes or for updating the prediction.

The introduction of the outbreak risk fundamentally differentiates our method from previous modelling approaches. The generalized linear model for Baden-Württemberg^29^ determined the infections at the district level from six predictors. Although its district-related scaling factor that accounted for differences in the local magnitude of PUUV-incidence did not have any straightforward mechanistic interpretation, it mirrored outbreak risk as it is used here. On the other hand, each of the two separate CART-based models for districts in Southern and Northern Germany^30^ directly determined the risk classes at the district level from four predictors. Those models demonstrated the benefit of a schematic representation of the model target and inspired our implementation of a geometrically-motivated classification algorithm.

Our prediction model does not require bank vole population data (e.g., abundance and PUUV-prevalence) and therefore does not require fieldwork such as monitoring, trapping and sampling. Nevertheless, such data are essential for validating the model, especially to minimize inaccurate predictions. In the future, it should be clarified how flowering intensity and seed production are related and which conditions lead to deviations in the causal chain rodent outbreak–PUUV-prevalence–human PUUV-incidence. Answers to such questions would be crucial for developing an updated version of the predictive model based on the present study, and for testing its conclusions and hypotheses.

## Methods

Data acquisition, processing and modelling was performed using Python version 3.8^40^. This study is based on our previous work that applied Support Vector Machines to develop a straightforward robust model for the human PUUV-infection risk at the district level^34^.

The analysis was limited to the PUUV-infections after 2006 to avoid bias due to underreporting in the early years when PUUV-infections became notifiable. The years 2006–2021 contained 91.9% of the total reported PUUV-cases from 2001 to 2021. The years 2022 and 2023 were used as an external test set. Each observation in the dataset represented a specific district in a specific year. The variables of the observations were the available predictors’ values in that district in that year. The labels of the observations were the PUUV-infections, incidence, and risk classes as well as the outbreak risk, a new quantity introduced by this study.

### Data acquisition and preparation

Table 3 shows the collected and digitized datasets, together with their source, the corresponding period for their time series, as well as their temporal and spatial resolution.

**Table 3:** The datasets used in this analysis.

Dataset	Source	Period/Year	Temporal resolution	Spatial resolution
Human PUUV-infections	SurvStat@RKI 2.0^33^	2006–2023	Annual (and weekly)	At the district level
Population	Eurostat^41^	2006–2021	Annual	At the district level
Weather data	Climate Data Center^42^	2004–2022	Monthly	1 × 1 km^2^ grid
Flowering intensity of the common beech	Dagmar Schneck (AFZ-DerWald^43–46^ and personal communication)	2005–2022	ANNUAL	At the level of the regions of provenance for the beech
Land cover data	CORINE Land Cover^47^	2018	Annual	100 × 100 m^2^ grid
Vector data for Germany's district boundaries	Federal Agency for Cartography and Geodesy^48^	2017	N/a	N/a

The training set contained the years 2006–2021. The external test set contained the years 2022 and 2023.

Human PUUV-incidence was calculated as the number of infections per 100,000 people, by using population data. For each year, we used the population reported for the January 1 of that year. The population of 2021 was also used for 2022 and 2023.

The collected datasets of weather parameters were: mean values of the mean daily air temperature in °C—Tmean, minimum daily air temperature in °C—Tmin, maximum daily air temperature in °C—Tmax, total precipitation in mm—Pr, total sunshine duration in hours—SD, mean soil temperature at 5 cm depth in °C—ST, mean soil moisture in percent plant useable water—SM. The grids for each of these datasets were processed using the vector layer of the district boundaries of Germany and the average values of the corresponding parameter were calculated at the district level.

### Combination of urban and rural districts

To compensate partially for potential differences between the reported place of residence and the place of infection in notification reports of human PUUV-infections, we combined most of the urban districts with their surrounding rural district (for details of the 12 such districts see^34^). Any combined district is explicitly mentioned in the text as appropriate for clarity. The only urban districts that remained separate were SK Köln (Cologne), SK Münster, and SK Stuttgart, whose areas are distinctly large. The naming convention matches that of the German version of SurvStat@RKI 2.0, where LK stands for a rural district (from the German “Landkreis”) and SK stands for an urban district (from the German “Stadtkreis”).

The observations for the combined districts retained the designation of the rural district. For the infections and populations, we aggregated the individual values and calculated the incidence. For the weather parameters, we assigned the mean values weighted by the area of each district. The calculation of the beech flowering intensity is described in a following section.

### Selection of the districts

**Figure 4:**
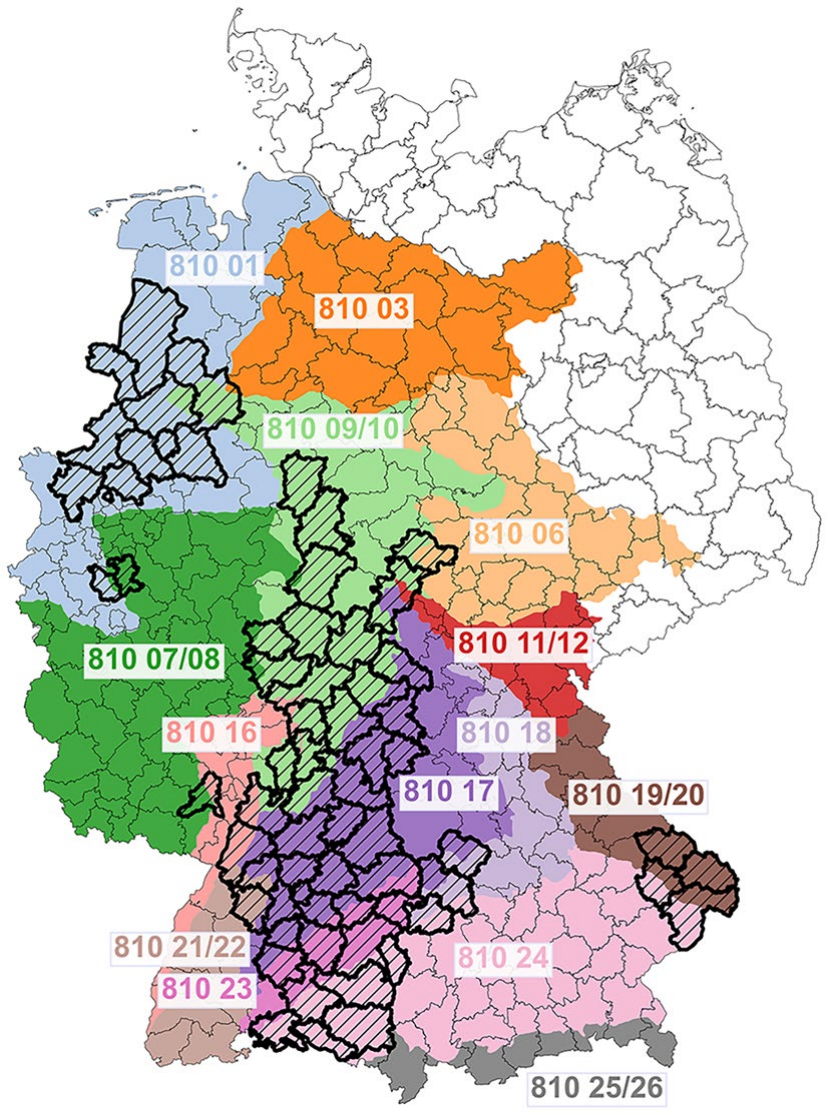
The districts included in the analysis are shown with thick borders and black hatching. The relevant regions of provenance (HKG) for the common beech are shown with different coloring. The HKG 02, 04, 05 and 13–15 in the northeast don’t matter for this analysis and are not shown for simplicity. Own representation, based on the map by the Federal Office for Agriculture and Food (BLE)^53^. The underlying map is from the Federal Agency for Cartography and Geodesy (BKG)^48^.

To select the districts where PUUV is endemic and to exclude individual cases from non-endemic regions, we included in the analysis only districts where the total infections were ≥ 20 and the maximum annual incidence was ≥ 2 in the period 2006–2021. A total of 66 districts were selected (Fig. 4) based on these criteria: 26 districts from Baden-Württemberg (BW), 16 from Bavaria (BY), 8 from Hesse (HE), 3 from Lower Saxony (NI), 10 from North Rhine-Westphalia (NW), 1 from Rhineland-Palatinate (RP), and 2 from Thuringia (TH). The selected districts accounted for 10,090 human PUUV-infections, 89.9% of the total infections reported countrywide during this period.

### Incidence transformation and outbreak risk

We applied a log-transformation to the incidence values^30^, to increase the impact of nonzero values. To consider the effects that drive the occurrence of high district-relative incidence, we then discretized the incidence at the district level with two bins, i.e., we binarized it. For the binarization, we performed an unsupervised clustering of the log-transformed incidence, separately for each district. This transformation compensated for the high spatial inhomogeneity of the incidence and converted its prediction into a problem of extrema detection.

The binary classification, which we call the “outbreak risk”, resulted in two classes labelled “low risk” (0) and “high risk” (1). A local “outbreak” occurred in a year when the incidence in a district was classified in the high-incidence bin of the past reported values. The incidence in districts for non-outbreak years was zero or considered low relative to the past reported values during an outbreak in that particular district. From the total 1056 observations (16 years × 66 districts), 682 were assigned low outbreak risk (65%) and 374 were assigned high outbreak risk (35%).

### Beech flowering intensity

The area of Germany is divided into 26 regions of provenance^49^ (abbreviated as HKG, from the German term “Herkunftsgebiet”) for the common beech characterized by similar ecological and geographical conditions that connect beech stands of the same provenance^50^ (Fig. 4). Characteristic tree stands are selected from each HKG and their flowering intensity is assessed^38,43^ once a year in the first weeks of June^51^. For the evaluation of the flowering intensity, a qualitative 4-class scale is used^43^. The individual evaluations are averaged over each HKG, and published in the August issue of the forestry magazine AFZ-DerWald^52^.

The time series for the flowering intensity (FI) at the HKG level in 2005–2022 were collected from personal communication with Dagmar Schneck from the State Office for Forest Reproductive Material, Brandenburg State Forestry Office, data that offer additional accuracy compared to the data published in AFZ-DerWald^43,45^. The FI for the combined HKG (07/08, 09/10, 11/12, 19/20, 21/22, and 25/26) correspond to the mean values of the individual regions of provenance. Our analysis was performed at the district level, a much finer spatial resolution than the HKG level of FI. Therefore, we approximated the FI by interpolating at the district level. To that end, for every district we calculated the weighted arithmetic mean of the flowering intensities from each HKG contained in the district ($F{I}_{{\text{HKG}}_{i}}$), with the proportions of its broad-leaved forest ($BF$)^54^ that belongs to each HKG (${P}_{{\text{HKG}}_{i}}$) as weights (Eq. 1). The broad-leaved forest was estimated from the homonymous class 3.1.1 of the Corine Land Cover (CLC) 2018, which includes areas with > 75% cover of deciduous and evergreen broad-leaved tree species, with pure or mixed stands of beech, alongside oak, hornbeam, lime, maple, ash, poplar, and birch among others^55^.

1 $$F{I_{{\text{district}}}} = \frac{{\mathop \sum \nolimits_i \left( {F{I_{{\text{HK}}{{\text{G}}_i}}} \cdot {P_{{\text{HK}}{{\text{G}}_i}}}} \right)}}{{\mathop \sum \nolimits_i {P_{{\text{HK}}{{\text{G}}_i}}}}},\,{\text{where}}\,{P_{{\text{HK}}{{\text{G}}_i}}} = \frac{{BF\;{\text{of district in HK}}{{\text{G}}_i}}}{{{\text{Total }}BF{\text{ of district}}}}$$

For the observations from combined districts, we calculated the $FI$ as the weighted arithmetic mean of the flowering intensities from each of the two combined districts, with the areas of the broad-leaved forest in each district as weights (Eq. 2).

2 $$\begin{aligned} F{I_{{\text{combined}}}} = &\frac{{F{I_{{\text{urban}}}} \cdot B{F_{{\text{urban}}}} + F{I_{{\text{rural}}}} \cdot B{F_{{\text{rural}}}}}}{{B{F_{{\text{urban}}}} + B{F_{{\text{rural}}}}}} \\ = & \frac{{F{I_{{\text{urban}}}} \cdot {A_{{\text{urban}}}} \cdot BF{\%_{{\text{urban}}}} + F{I_{{\text{rural}}}} \cdot {A_{{\text{rural}}}} \cdot BF{\%_{{\text{rural}}}}}}{{{A_{{\text{urban}}}} \cdot BF{\%_{{\text{urban}}}} + {A_{{\text{rural}}}} \cdot BF{\%_{{\text{rural}}}}}}\end{aligned},{\text{ where}}\,A{\text{ is the district area}}$$

### Classification method and incidence prediction

We concentrated only on those combinations of predictors that led to a linear decision boundary (hyperplane) for the classification of the outbreak risk. To detect the outbreaks, we used a classifier based on a SVM with a linear kernel, because this algorithm combines high performance with low model complexity, by returning the decision boundary as a linear equation of the inputs.

**Figure 5:**
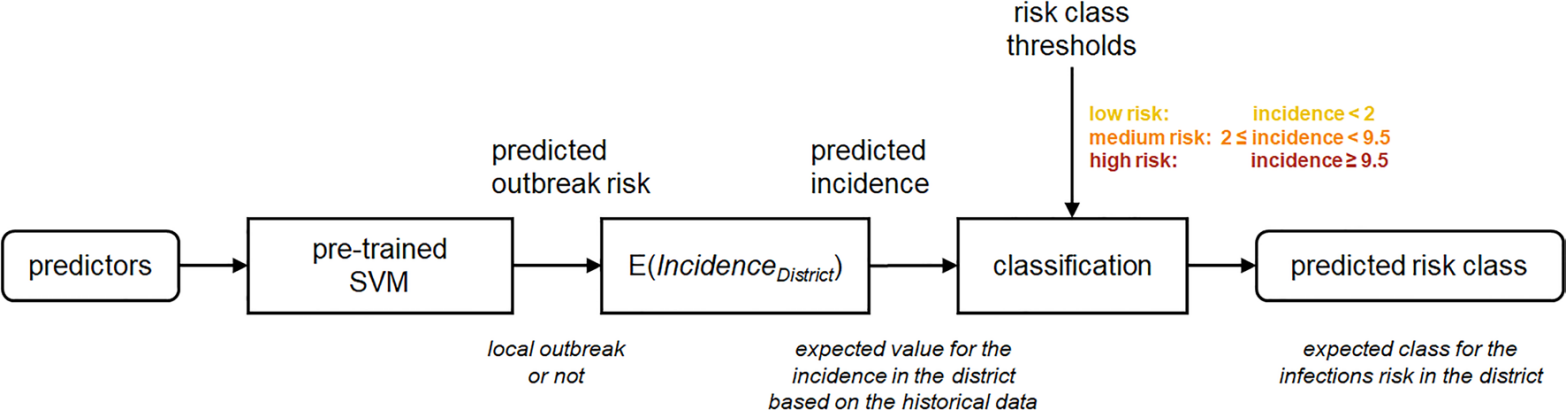
Schematic representation of the prediction method, after training the SVM classifier.

Figure 5 shows the complete prediction model. The output of the SVM represents the expected outbreak risk in each district: a 0 (low outbreak risk, i.e., no expected local outbreak) or a 1 (high outbreak risk, i.e., expected local outbreak). Our analysis assumes that the annual incidence in each district is fluctuating between two extreme values: a low value corresponding to the class of low outbreak risk, and a high value corresponding to the class of high outbreak risk. The incidence for each class of outbreak risk follows a probability distribution, which is sampled by the historical data. Assuming that the probabilities of both classes follow a gamma distribution, the outbreak risk is converted to an incidence value by calculating the mean incidence from all observations in the training dataset from the district, which had the same outbreak risk, either low (0) or high (1).

The predicted risk class is determined by a simple classification of the predicted incidence by applying predefined thresholds. We selected three risk classes, defined by slight drops in the histogram of the past incidence values. The first threshold of 2 infections per 100,000 population limits the low-risk class. The second threshold, which separates the medium-risk and the high-risk classes, was set at 9.5 infections per 100,000 population.

### Feature selection

The performance of each SVM model was evaluated using two methods:

- the explanatory power assessed the model performance on the entire training dataset, and
- the predictive power estimated the method performance on a test dataset.

The explanatory power was a fast computation after the model training. It was used to restrict the search space, for performing the more accurate but intensive computation of the predictive power. The latter was evaluated by a leave-one-out cross-validation (LOOCV): a single year was removed from the training dataset (2006–2021), then the model was trained using the remaining years, and this trained model was used to predict the incidence values for the removed year; this was repeated for all sixteen years in the training dataset; finally, the prediction for all years was evaluated using the accuracy of the outbreak risk.

From each monthly weather parameter, 21 entries were created for all months of the previous two years until the previous September, as possible predictors for the model. In total, there were 147 weather predictors (7 weather parameters × 21 months). The two final possible predictors were the beech flowering intensity from the previous year and from two years before. The available predictors are thereafter called “variables”.

A model with all 149 predictors (147 weather variables + 2 flowering intensity variables) had an accuracy for the outbreak risk in the training dataset (explanatory power) of 89.5%. On the other hand, its mean annual accuracy according to the LOOCV (predictive power) was only 49.1%, which was considered extremely low and was evidence of overfitting that is expected with a large number of predictors that are highly correlated with each other. Limiting the number of predictors for our final model reduced the risk of overfitting. Therefore, we established a heuristic method to limit and efficiently search the multidimensional variable space.

We first applied a one-dimensional SVM with a linear kernel for each of the two flowering intensity variables. The performance of the model with the flowering intensity of the previous year was better than that of the model with the flowering intensity from two years before (accuracy 79.6% vs. 73.2%). Since the two variables had a strong negative correlation (− 0.72 Pearson correlation coefficient, p ≪ 0.001), the flowering intensity from two years before was removed from our pool of predictors and only the flowering intensity of the previous year remained.

For the further search, we focused on subsets with up to 6 weather predictors. The flowering intensity of the previous year was considered separately in order to quantitatively assess its influence. We applied an SVM with a linear kernel for all 2-variable combinations of all 147 possible weather variables ($\frac{147!}{2!\cdot\left(147\text{-}2\right)!} =10{,}731$ combinations). We selected the year-month combinations with the most occurrences in the best 100 models. To avoid sets with highly correlated variables, we formed the further subsets with N variables, by selecting the N-variable combinations, with exactly one variable from each of the N best year-month combinations (N-fold Cartesian product) for models without or with the flowering intensity of the previous year. From all the models for each different number of weather variables up to 6, we selected the model with the best explanatory power. Finally, using LOOCV, we assessed the predictive power of the 13 optimal models: 6 models with only weather variables and 7 models including the beech flowering intensity of the previous year.

## Data Availability

The data that support the findings of this study are available from the corresponding author upon reasonable request.

## Code availability

We performed data acquisition, processing, analysis and visualization using Python version 3.8 with the following packages: Requests, Selenium, Beautiful Soup, Numpy, Pandas, Geopandas, Shapely, Rasterio, scikit-learn, SciPy, Matplotlib and seaborn. The support vector machine classifier was trained with the algorithm SVC from the sklearn.svm module. The code that supports the findings of this study is available from the corresponding author upon reasonable request.
